# Epidemiology of hospitalizations due to pesticide intoxication-associated acute kidney injury in China

**DOI:** 10.1007/s11255-021-02885-9

**Published:** 2021-05-18

**Authors:** Min He, Yanhua Wu, Zhen Xie, Zhilian Li, Li Hao, Guohui Liu, Qiang He, Yuanjiang Liao, Rizwangul Ghappar, Hongwei Peng, Jinglie Xie, Xiaohong Wei, Yusheng Zhang, Qiongyi Huang, Yuanhan Chen, Xinling Liang, China collaborative study on AKI (CCS-AKI)

**Affiliations:** 1 The Second School of Clinical Medicine, Southern Medical University, Guangzhou, 510515 China; 2 Division of Nephrology, Guangdong Provincial People’s Hospital, Guangdong Academy of Medical Sciences, 106 Zhongshan Road 2, Guangzhou, 510080 China; 3 Division of Nephrology, Yue Bei People’s Hospital Affiliated to Shantou University Medical College, Shaoguan, 512026 China; 4 Guangdong Provincial Geriatrics Institute, Guangzhou, 510080 China; 5 Department of Dermatology, Sichuan Provincial People’s Hospital, University of Electronic Science and Technology of China, Chengdu, 610072 China; 6 Department of Nephrology, Second Hospital of Anhui Medical University, Hefei, 230601 China; 7 Department of Nephrology, Dongguan People’s Hospital, Dongguan, 523018 China; 8 Department of Nephrology, Zhejiang Provincial People’s Hospital (People’s Hospital of Hangzhou Medical College), Hangzhou, 310014 China; 9 Department of Nephrology, Chongqing Ninth People’s Hospital, Chongqing, 400700 China; 10 Department of Nephrology, First People’s Hospital of Kashgar, Kashgar, 844000 China; 11 Department of Nephrology, People’s Hospital of Wanning, The First Affiliated Hospital of Chongqing Medical University, Wanning, 571500 China; 12 Department of Nephrology, Lufeng People’s Hospital, Shanwei, 516500 China; 13 Department of Nephrology, Chongzuo People’s Hospital, Chongzuo, 844000 China; 14 Second Division of Internal Medicine, Wuhua People’s Hospital, Meizhou, 514400 China

**Keywords:** Acute kidney injury, Pesticide, Epidemiology, Risk factor, Adverse outcome

## Abstract

**Background:**

There is a paucity of epidemiological data regarding pesticide intoxication-associated acute kidney injury (AKI). Therefore, the aim of this study was to identify the epidemiological features, risk factors, and adverse outcomes of AKI in this population.

**Methods:**

The data used in this multi-center, hospitalized population-based, retrospective study were retrieved from electronic medical records. AKI was defined as an acute increase in serum creatinine according to the criteria of Kidney Disease: Improving Global Outcomes. The Charlson Comorbidity Index was used to evaluate the burden of in-hospital mortality.

**Results:**

Of 3,371 adult patients in 11 hospitals, 398 (11.8%) were diagnosed with AKI (grade 1, 218 [6.5%]; grade 2, 89 [2.6%]; grade 3, 91 [2.7%]). Herbicide intoxication was associated with the highest incidence of AKI (53.5%) and higher grades of AKI. After multivariable adjustment, pesticide categories and moderate or severe renal disease were independently associated with AKI. As compared with the referred category, insecticide and herbicide intoxications were associated with a 1.3-fold (95% CI 1.688–3.245) and 3.8-fold (95% CI 3.537–6.586) greater risk of AKI. Regardless of the pesticide category, AKI was independently associated with in-hospital mortality, with odds ratios of 3.433 (95% CI 1.436–8.203) for insecticides, 2.153 (95% CI 1.377–3.367) for herbicides, and 4.524 (95% CI 1.230–16.632) for unclassified or other pesticides.

**Conclusion:**

AKI is common in pesticide intoxication and associated with an increased in-hospital mortality. Herbicides pose the greatest risks of AKI and death.

## Introduction

As a global public health concern, acute kidney injury (AKI) is not only associated with in-hospital adverse outcomes, but also increased short- and long-term risks of cardiovascular disease, chronic kidney disease, and mortality [1]. The International Society of Nephrology proposed the 0by25 program that aims to prevent all avoidable deaths due to AKI by the year 2025 [2]. Although the worldwide incidence of AKI continues to increase, the epidemiology and treatment outcomes differ by regions and economic factors [3, 4]. The implementation of measures for the prevention of AKI is a major challenge in low-resource and low-income regions.

Pesticides, including herbicides, insecticides (which may include insect growth regulators, termiticides, etc.), nematicides, molluscicides, piscicides, avicides, rodenticides, bactericides, insect repellents, animal repellents, antimicrobials, and fungicides, are chemical compounds that are meant to control pests. Pesticides are widely used to promote economic development of agriculture-dependent countries in the Asia–Pacific region. However, the application of pesticides also threatens human health. Pesticide exposure can cause injury to the skin, as well as the nervous, reproductive, gastrointestinal, and urinary systems. Epidemiological studies have reported that long-term pesticide exposure is associated with chronic renal injury [5, 6]. For example, the Agricultural Health Study, a prospective study of cancer and other health outcomes, found that licensed pesticide applicators and their spouses are at higher risk of end-stage renal disease [7, 8]. Pesticide applicators in the Asia–Pacific region are also at greater risks of social problems, especially suicide [9–11]. A report by the World Health Organization estimated that pesticides were associated with 186,000 preventable suicides and 4.42 million disability-adjusted life years annually [12].

There is a paucity of epidemiological data of pesticide intoxication-associated AKI. According to a recent report based on the Taiwan Health Insurance Research Database, organophosphate intoxication was associated with a long-term higher risk of AKI [13]. Considering the acute nature of pesticide intoxication, the short-term effects on AKI also poses risk to human health. In addition, only organophosphate intoxication was investigated in the Taiwan study, thus the effects of different categories of pesticides should be further compared.

Therefore, the aim of the present multi-center retrospective study was to determine the epidemiological features, risk factors, and short-term adverse outcomes of adult patients with pesticide intoxication-associated AKI.

## Methods

### Study design and data sources

The China Collaborative Study on Acute Kidney Injury (CCS-AKI), sponsored by the Guangdong Provincial People’s Hospital (Guangzhou, Guangdong Province, China), was a multi-center, hospitalized population-based, retrospective study designed to identify the epidemiological features of AKI in various regions and clinical settings. Demographic characteristics, diagnoses, and AKI-related laboratory test results were derived from electronic medical records. Creatinine measurements were adjusted to account for intrahospital differences. The CCS-AKI included 19 hospitals.

The CCS-AKI is registered with the clinicaltrials.gov website under registration number NCT03054142. The study protocol was approved by the Ethics Research Committee of Guangdong Provincial People’s Hospital (approval no. GDREC2016327H) and conducted in accordance with the ethical principles for medical research involving human subjects as stated in the Declaration of Helsinki.

### Clinical definitions

AKI was defined as an increase in serum creatinine of 0.3 mg/dL within 48 h or a 50% increase from baseline within 7 days based on the criteria of Kidney Disease: Improving Global Outcomes [14]. The stage of AKI was determined using the peak creatinine level after AKI onset. Pesticides were classified into four categories: insecticides, herbicides, rodenticides, and unclassified or others.

The Charlson Comorbidity Index (CCI) was used to evaluate the burden of in-hospital mortality. Because the International Classification of Disease Code for CCS-AKI was not standardized among the participating hospitals, the CCI was calculated based on the diagnosis at discharge retrieved from electronic medical records [15]. According to the CCI definition, serum creatinine levels of > 3 mg/dL is regarded as a marker of moderate to severe renal disease [16].

### Statistical analyses

Non-normally distributed continuous variables are presented as medians (25th and 75th percentiles). Count data are expressed as the number of cases (%), and the difference between groups was compared using the Chi-squared test or Fisher’s exact test, as appropriate. The Bonferroni test was used for multiple comparisons of pesticide categories and AKI grades. Conditional multivariable logistic regression models were used to identify independent associations among variables. The associated 95% confidence intervals (CIs) were estimated. An interaction term was applied to estimate the effect of the pesticide category on AKI-associated mortality. If there was an interactive effect between the pesticide category and mortality, subgroup analysis was performed. All statistical analyses were performed using IBM SPSS Statistics for Windows, version 24.0. (IBM Corporation, Armonk, NY, USA). A two-tailed probability (*p*) value of < 0.05 was considered statistically significant.

## Results

### Clinical characteristics

The medical records of 3839 patients in 11 hospitals were retrieved from the CCS-AKI database, which included 50 cases of pesticide intoxication. According to the Institute of Geographic Sciences and Natural Resource Research (Chinese Academy of Sciences, Beijing, China), these 11 hospitals cover five of nine agricultural regions in China [17], including Southern China, the Yunnan-Guizhou Plateau, Northern arid and semiarid region, Sichuan Basin and surrounding regions, and Middle-lower Yangtze Plain. After exclusion of patients aged < 18 years, 3371 adult patients (mean age 41 years; age range 28–54 years; 1470 [43.6%] males) were included in this study. Among them, 1214 (36.0%), 989 (29.3%), 512 (15.2%), and 598 (17.7%) were treated for intoxication with insecticides, herbicides, rodenticides, and unclassified or other pesticides, respectively, and 58 (1.7%) were treated for exposure to multiple pesticides.

AKI was detected in 398 (11.8%) patients (grade 1, 218 [6.5%]; grade 2, 89 [2.6%]; grade 3, 91 [2.7%]). Compared with those without AKI, patients with AKI had higher rates of peripheral vascular disease, cerebrovascular disease, moderate or severe renal disease, and liver disease, as well as higher CCI scores (Table 1).

**Table 1:** Clinical characteristics by acute kidney injury

	Non-AKI	AKI	*χ*^2^/U	*p* value
Gender, male	1292 (43.5%)	177 (44.5%)	0.143	0.706
Age, years	41 (29, 54)	40 (28, 55)	− 0.342	0.733
Pesticides categories			147.053	< 0.001
Insecticides	1087 (89.5%)	127 (10.5%)		
Herbicides	775 (78.4%)	213 (21.6%)		
Rodenticides	492 (96.1%)	20 (3.9%)		
Other or unclassified	566 (94.6%)	32 (5.4%)		
Multiple	52 (89.7%)	6 (10.3%)		
Comorbidities				
Myocardial infarction	5 (0.2%)	2 (0.5%)	1.892	0.169
Congestive heart failure	95 (3.2%)	8 (2%)	1.668	0.197
Peripheral vascular disease	56 (1.9%)	14 (3.5%)	4.604	0.032
Cerebrovascular disease	67 (2.3%)	16 (4%)	4.555	0.033
Dementia	8 (0.3%)	2 (0.5%)		0.334*
Chronic pulmonary disease	47 (1.6%)	10 (2.5%)	1.83	0.176
Connective tissue disease	16 (0.5%)	5 (1.3%)		0.093*
Peptic ulcer disease	13 (0.4%)	3 (0.8%)	0.743	0.389
Mild liver disease	106 (3.6%)	24 (6%)	5.744	0.017
Diabetes without end-organ damage	64 (2.2%)	11 (2.8%)	0.601	0.438
Hemiplegia	2 (0.1%)	0 (0%)		1*
Moderate or severe renal disease	98 (3.3%)	81 (20.4%)	202.979	< 0.001
Diabetes with end-organ damage	3 (0.1%)	1 (0.3%)		0.395*
Tumor without metastasis	26 (0.9%)	2 (0.5%)		0.766*
Leukemia	12 (0.4%)	1 (0.3%)		1*
Lymphoma	1 (0%)	0 (0%)		1*
Moderate or severe liver disease	4 (0.1%)	2 (0.5%)		0.151*
Metastatic solid tumor	42 (1.4%)	10 (2.5%)	2.792	0.095
AIDS	3 (0.1%)	0 (0%)		1*
Any liver disease	107 (3.6%)	25 (6.3%)	6.704	0.01
Any diabetes	65 (2.2%)	11 (2.8%)	0.53	0.467
Any malignant disease	74 (2.5%)	13 (3.3%)		0.398*
Score of Charlson Comorbidity Index	0 (0, 0)	0 (0, 2)	− 9.484	< 0.001

*The significance was calculated by Fisher's exact test

### AKI in different pesticide categories

**Fig. 1:**
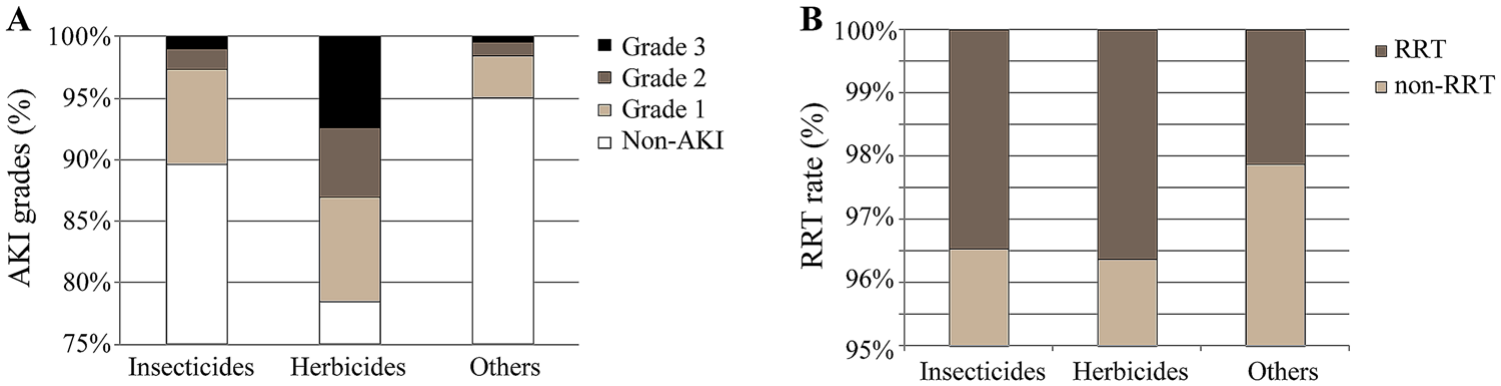
AKI by pesticide categories. **a** AKI incidences and grades. Multiple comparisons among groups using Bonferroni correction: the incidence of AKI grade 2 or 3 was higher with herbicides than the other two groups, while comparable between the other two. **b** Rates of renal replacement therapy. The rates of renal replacement therapy were comparable among the three groups (*χ*^2^ 5.112, *p* = 0.078). *AKI* acute kidney injury, *RRT* renal replacement therapy

Among the hospitalized patients, the highest incidence of AKI (53.5%) was associated with herbicide intoxication, following by insecticide intoxication (Table 1). The relationships among pesticide categories and AKI severity were further analyzed. Because of the limited number of cases, analysis was limited to the categories of insecticides, herbicides, and rodenticides. Besides the highest incidence, herbicide intoxication was associated with higher grades of AKI. For intoxication with herbicides, insecticides, and other pesticides, the rates of grade 2 AKI were 5.7%, 1.6% and 1.1%, and the rates of grade 3 were 7.4%, 1.1% and 0.4%, respectively (Fig. 1a). A total of 103 patients (3.1%) underwent renal replacement therapy (RRT). The rates of RRT were comparable among patients poisoned by insecticides, herbicides, and rodenticides (3.5%, 3.6%, and 2.1%, respectively, *p* = 0.078) (Fig. 1b).

### Risk factors for AKI

To assess the risk factors for AKI, baseline variables that were considered clinically relevant or with a univariate relationship with AKI were entered into a multivariable logistic regression model (in stepwise forward conditional mode). These variables were pesticide categories (non-insecticides and non-herbicides as reference), age, peripheral vascular disease, cerebrovascular disease, any liver disease, and moderate or severe renal disease. After adjustment for multiple variables, pesticide categories and moderate or severe renal disease were independently associated with AKI development (Table 2). As compared with the referred category, insecticide and herbicide intoxications were associated with a 1.3-fold (95% CI 1.688–3.245) and 3.8-fold (95% CI 3.537–6.586) greater risk of AKI, respectively.

**Table 2:** Risk factors for AKI

	Odds ratio	95% confidence interval	*p* values
Pesticides category			< 0.001
Insecticides	2.340	1.688–3.245	< 0.001
Herbicides	4.826	3.537–6.586	< 0.001
Moderate or severe renal disease	6.490	4.658–9.042	< 0.001

The variables selected into multivariable logistic regression model for association analysis of AKI (in stepwise forward conditional mode) were pesticides category (non-insecticides and non-herbicides as reference), age, peripheral vascular disease, cerebrovascular disease, any liver disease and moderate or severe renal disease

### Association between AKI and in-hospital mortality

**Fig. 2:**
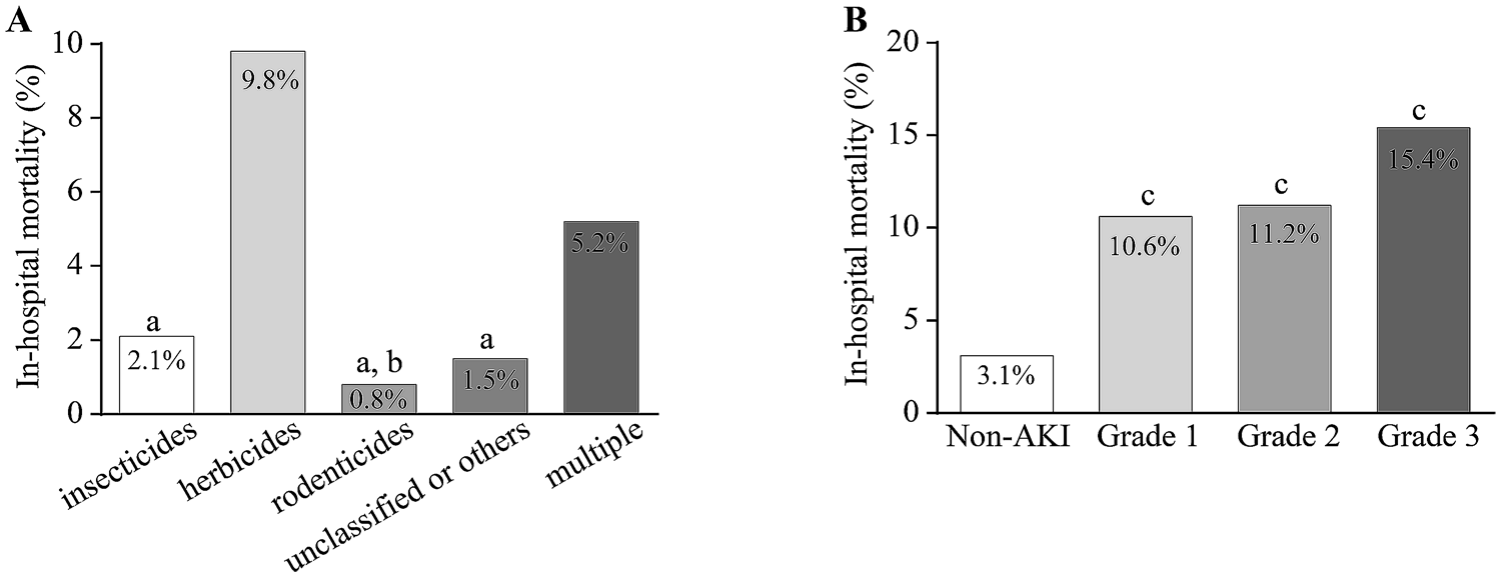
In-hospital mortality in clinical settings. **A** The in-hospital mortality rate differed significantly among the different pesticide categories. a Significant difference vs. herbicides; b: significant difference vs. multiple pesticides. **B** The in-hospital mortality rate was higher for any grade of AKI as compared with non-AKI. However, the mortality rates were comparable among various grades of AKI. Bonferroni adjustment was used for multiple comparisons.

A total of 139 patients (4.1%) died during hospitalization. The raw in-hospital mortality rates for intoxications with herbicides, multiple pesticides, insecticides, unclassified or other pesticides, and rodenticides were 9.8%, 5.2%, 2.1%, 1.5%, and 0.8%, respectively (Fig. 2a). After Bonferroni adjustment, the mortality rate associated with herbicides was higher than that of insecticides, unclassified or other pesticides, and rodenticides, and the mortality rate associated with multiple pesticides was higher than that of rodenticides. The mortality rates for non-AKI, and grades 1, 2, and 3 AKI were 3.1%, 10.6%, 11.2%, and 15.4%, respectively. By Bonferroni adjustment for multiple comparison, the mortality rate was higher for all AKI grades than non-AKI. Although the results were not significant among the various grades of AKI, this increasing tendency indicated that the risk of mortality gradually increased with a higher grade of AKI (Fig. 2b).

Because of an interaction between AKI and the pesticide category related to mortality in the preliminary analysis, the independent association of AKI with mortality was further assessed by stratification of the pesticide categories. Regardless of the category of pesticides, AKI was independently associated with in-hospital mortality, with odds ratios of 3.433 (95% CI 1.436–8.203) for insecticides, 2.153 (95% CI 1.377–3.367) for herbicides, and 4.524 (95% CI 1.230–16.632) for unclassified or other pesticides (Table 3).

**Table 3:** Association between AKI and in-hospital mortality

	Odds ratio	95% CI	*p* value
Insecticides			
CCI	1.223	1.007–1.485	0.042
University hospital	12.091	1.617–90.425	0.015
AKI	3.433	1.436–8.203	0.006
Herbicides			
University hospital	6.247	1.948–20.033	0.002
AKI	2.153	1.377–3.367	0.001
Other or unclassified pesticides			
CCI	1.458	1.167–1.822	0.001
AKI	4.524	1.230–16.632	0.023

Variables of CCI, age, university hospital and AKI were selected into the multivariable logistic regression model in a forward conditional mode

*AKI* acute kidney injury, *95% CI* 95% confidential interval, *CCI* Charlson Comorbidity Index

## Discussion

The aim of this multi-center investigation was to identify the epidemiological features of pesticide intoxication-associated AKI. The results showed that the AKI was common in this population. Herbicides, the second most frequently applied pesticide, was associated with the highest risk and grade of AKI. After adjustment for multiple variables, herbicide intoxication was associated with a 3.8-fold greater risk of AKI. The in-hospital death rate was highest for herbicide intoxication. By stratification of pesticide categories, multivariable analysis identified an independent association of AKI with in-hospital mortality.

Pesticide intoxication remains an important public health concern, especially in Asia. According to national data of South Korea, the age-standardized rate of pesticide intoxication was 15.37 per 100,000 persons between 2006 and 2010, of which 57.3% of cases required hospitalization [18]. To the best of our acknowledgement, this is the first study to evaluate the epidemiological features of AKI in this population. The raw incidence of AKI of 11.8% in this study was similar with the overall incidence of 11.6% in the EACH study (Epidemiology of AKI in Chinese Hospitalized Adults) [19]. However, the CCS-AKI criteria for AKI classification in the present study, which differed from those of the EACH study, strictly followed the definition of Kidney Disease: Improving Global Outcomes, whereas extended criteria were applied in the EACH study, which may have resulted in a higher incidence of AKI. A recent pilot study of CCS-AKI conducted by our group showed that the overall incidence of AKI in hospitalized adults was 8.2% and middle-aged patients were at the lowest risk of AKI [20]. Considering that most patients hospitalized for pesticide intoxication were middle-aged, the risk of AKI in this population seemed to be higher than the overall hospitalized population. Notably, only hospitalized patients were included for analysis, thus the resulting epidemiological features in this study cannot necessarily be applied to other populations.

Although the detailed mechanisms have not been clarified, previous animal studies have demonstrated that pesticides can directly cause renal injury through reductions in metabolic functions and induction of oxidative stress [21–23]. Pesticide intoxication also manifests as gastrointestinal symptoms and gastric salvage further exacerbates fluid loss. Among the various categories of pesticides, herbicide intoxication is associated with greater risks of both AKI and mortality. From this perspective, herbicides are the most toxic. To the best of our knowledge, this study is the first to compare the renal toxic effects among various pesticide categories.

The strengths of this study include the large study sample. Thus, multiple comorbidities were adjusted for subgroup analysis to control for confounders. The use of multi-center data acquired from multi-level hospitals is another advantage. China is a large developing country that is going through industrial transformation from a typical agriculture country. Imbalances in economic and medical developments are evident across the nation. Thus, the epidemiological features of AKI associated with pesticide intoxication in China will provide valuable information for other developing countries. Among the 19 hospitals in the phase 1 database of CCS-AKI, four hospitals in first-tier cities (Beijing, Shanghai, Guangzhou, and Shenzhen) and four tertiary hospitals in capital cities (Hohhot, Urumqi, and Changchun) were excluded because of the low numbers of patients with pesticide intoxication, indicating that pesticide intoxication is not necessarily an important issue in developed industrial cities, but remains significant in low-resource agricultural regions.

Pesticide intoxication is an emergency condition. Although the diagnostic and therapeutic capabilities of local and non-university hospitals are often inadequate, as revealed by a recent survey [24], the benefit of referral to higher-level hospitals remains controversial. In this study, four of the 11 participating hospitals were province-level central hospitals and university hospitals. However, treatment in these hospitals was associated with a greater risk of death as compared with non-university hospitals (Table 3), suggesting that referral does not necessarily improve survival. This result might be attributed to the poorer condition of patients in central hospitals. While unavoidable delays for referral should also be considered, local availability of treatment is a reasonable strategy. Thus, improvement in local medical facilities is urgently needed.

Because of the retrospective study design, it was not possible to differentiate the indication of RRT for severe AKI, pesticide clearance, or multiple organ failure. Thus, a comparable rate of RRT (Fig. 1b) does not imply a similar rate of critical AKI among patients poisoned by different pesticides. Based on the finding that herbicide poisoning was associated with the highest rates of grade 2 and 3 AKI (Fig. 1a), herbicides seem to be the most detrimental pesticide with regard to AKI. Further, information regarding the exposure pathway and dose, severity of intoxication, and detoxification treatment was not available. In addition, it was not possible to determine whether the intoxication was self-induced, which is an important factor to improve survival. Thus, future prospective studies are needed to confirm these results.

In conclusion, AKI is a common complication of pesticide intoxication and associated with increased in-hospital mortality. Herbicides were associated with the highest risk of AKI and death.
